# Graphene quantum dots enhance the osteogenic differentiation of PDLSCs in the inflammatory microenvironment

**DOI:** 10.1186/s12903-023-03026-7

**Published:** 2023-05-27

**Authors:** Wanshan Gao, Yan Liang, Dongyan Wu, Sicheng Deng, Rongmin Qiu

**Affiliations:** 1 College of Stomatology, Hospital of Stomatology Guangxi Medical University , Guangxi Key Laboratory of Oral and Maxillofacial Rehabilitation and Reconstruction, Guangxi Clinical Research Center for Craniofacial Deformity, Guangxi Key Laboratory of Oral and Maxillofacial Surgery Disease Treatment, Guangxi Health Commission Key Laboratory of Prevention and Treatment for Oral Infectious Diseases, Nanning, 530021 Guangxi China; 2 Key Laboratory of Research and Application of Stomatological Equipment College of Stomatology Hospital of Stomatology Guangxi Medical University, Education Department of Guangxi Zhuang Autonomous Region, Nanning, Guangxi, China 530021

**Keywords:** Graphene quantum dots, Periodontal ligament stem cells, Osteogenic differentiation, Wnt/β-catenin signalling pathway

## Abstract

**Background and objective:**

Graphene quantum dots (GQDs), a type of carbon-based nanomaterial, have remarkable biological, physical, and chemical properties. This study investigated the biological mechanisms of the proliferation and osteogenic differentiation of human periodontal ligament stem cells (PDLSCs) induced by GQDs in an inflammatory microenvironment.

**Materials and methods:**

PDLSCs were cultured in osteogenic-induced medium with various concentrations of GQDs in standard medium or medium mimicking a proinflammatory environment. The effects of GQDs on the proliferation and osteogenic differentiation activity of PDLSCs were tested by CCK-8 assay, Alizarin Red S staining, and qRT‒PCR. In addition, Wnt/β-catenin signalling pathway-related gene expression was measured by qRT‒PCR.

**Results:**

Compared with the control group, the mRNA expression levels of ALP, RUNX2, and OCN and the number of mineralized nodules were all increased in PDLSCs after treatment with GQDs. Moreover, during the osteogenic differentiation of PDLSCs, the expression levels of LRP6 and β-catenin, which are Wnt/β-catenin signalling pathway-related genes, were upregulated.

**Conclusion:**

In the inflammatory microenvironment, GQDs might promote the osteogenic differentiation ability of PDLSCs by activating the Wnt/β-catenin signalling pathway.

## Introduction

Chronic periodontitis (CP) is an inflammatory and destructive disease invading the integrity of periodontal support tissues and is considered to be the most common cause of tooth loss in adults [1, 2]. Due to the role of periodontal inflammation, periodontal tissue cannot be regenerated completely once it is destroyed, consequently resulting in the absorption of alveolar bone. Unfortunately, traditional periodontal therapy usually fails to restore alveolar bone [3, 4]. Therefore, it is necessary to develop effective methods to reverse the microenvironmental inflammation of the periodontium and achieve periodontal regeneration of cementum-PDL complexes and alveolar bone. Tissue engineering and stem cell technology thus represent a research hotpot, as it has been deemed the most ideal therapeutic method for the reconstruction of bone defects in recent decades [5, 6]. Periodontal tissue engineering aims to develop biological substitutes to restore, maintain, or improve tissue function by using a combination of stem cells, scaffolds, and suitable biochemical factors [7].

Human periodontal ligament stem cells (PDLSCs), a type of mesenchymal-like stem cell, have been isolated from periodontal ligament tissue and have attracted widespread attention as promising “seed cells” for the regeneration and reconstruction of periodontal tissue [7, 8]. Compared with mesenchymal stem cell (MSC) acquisition, the procedure of PDLSCs acquisition is simpler and more noninvasive [9]. It has been verified in subsequent studies that PDLSCs have multiple advantages, such as high proliferation pluripotency and self-renewal, and PDLSCs can also differentiate into osteoblasts, adipocytes, and cementoblasts both in vitro and in vivo [10]. In addition, the osteogenic differentiation capacity of PDLSCs, which is an important biological behaviour, could promote the regeneration of lost alveolar bone induced by periodontitis. However, the inflammatory microenvironment impairs the differentiation potential of PDLSCs [11]. It was reported that the expression of tumour necrosis factor-alpha (TNF-α) generally suppresses osteogenesis and increases among the various chronic inflammatory bone diseases, such as chronic periodontitis [12]. More specifically, TNF-α expression levels in the gingival tissue and serum of patients with periodontitis are higher than those of the healthy control group [13–15]. Therefore, it is still an important goal to explore new regeneration strategies for periodontal support tissue.

Since its obtention by Novoselov and Geim in 2004, graphene and its derivatives have attracted widespread public attention and become a hotspot for research in relevant areas for their applications in biomedicine, such as drug/gene delivery, biosensors, and tissue engineering [16, 17]. Graphene quantum dots (GQDs), as an important family of engineered nanomaterials, possess many properties, including quantum confinement and edge effects. Furthermore, due to their quasizero-dimensional structure, GQDs have many advantages in biomedical applications of tissue engineering, such as low toxicity, biocompatibility, optimal stability, and antibacterial activity both in vitro and in vivo when used at certain doses [18–20]. The ability of GQDs to promote MSC osteogenic differentiation has recently been highlighted [21, 22]. Wang et al. constructed an effective GQDs loading system based on layered double hydroxide (LDH) nanoparticles, which exhibit anti-inflammatory ability for potential integration with native bone tissue [23]. It was found that graphene oxide quantum dots (GOQDs) rather than GO could strongly induce osteogenic differentiation of stem cells isolated from human exfoliated deciduous teeth (SHED) [24]. It was demonstrated in our previous study that GQDs could accelerate osteoblastic differentiation and fibroblast differentiation of PDLSCs in the absence of osteogenic inducers (not yet published). However, due to the inflammatory microenvironment suppressing the osteogenic capacity of local stem cells, whether GQDs promote osteogenic differentiation of PDLSCs under periodontitis and the related underlying mechanisms are still unknown.

The Wnt/β-catenin signalling pathway, which is dependent on the function of β-catenin and defined as the canonical Wnt signalling pathway, was found to be highly involved in some aspects of bone growth and formation [25, 26]. There are a series of activators, receptors, inhibitors, and other modulators involved in bone formation through various processes, such as stem cell regeneration and osteoblast proliferation induction [27]. When the pathway is activated, β-catenin protein stably accumulates in the cytoplasm and then enters the nucleus and stimulates the transcription of downstream genes [28–30]. Currently, it is generally believed that the Wnt/β-catenin signalling pathway is involved in PDLSCs osteogenic differentiation [31]. A previous study reported that graphene participates in Wnt/β-catenin signalling pathway-mediated osteogenic differentiation by upregulating β-catenin expression [20, 23]. Accordingly, we deduced that GQDs might activate PDLSCs through the Wnt/β-catenin signalling pathway.

Therefore, based on our previous study, we further explored the effects of GQDs on the proliferation and osteogenic differentiation of PDLSCs in vitro, and the underlying molecular mechanisms were further explored. Our hypothesis was that in the inflammatory microenvironment, GQDs could induce and enhance the osteogenic differentiation of PDLSCs by stimulating the Wnt/β-catenin signalling pathway.

## Materials and methods

### Cell culture

PDLSCs were isolated from healthy premolars extracted for orthodontic purposes from young patients aged 12–18. First, the primary PDLSCs were cultured by the tissue block method at 37 °C in a humidified atmosphere of 5% CO_2_ for 5 h. Premolars were rinsed in phosphate-buffered saline (PBS) containing penicillin/streptomycin antibiotic solution (Wisent, Canada), and the periodontal ligament in the middle of the root was gently scraped and cut into 1 mm^3^ sections with a sterile blade. Then, the sections were arrayed at the bottom of a cell culture flask with DMEM-F12 (Wisent, Canada) containing 20% FBS and 1% penicillin/streptomycin antibiotics, which were renewed every three or five days. When the cells reached 80% confluence, they were digested with 0.25% trypsin (Wisent, Canada) and passaged to obtain primary PDLSCs. Second, primary PDLSCs were isolated by the limited dilution cloning method. PDLSCs at the 3rd passage (P3) were used in the subsequent experiments.

### Flow cytometry

PDLSCs were digested and suspended in PBS at a concentration of 100 w/ml. Five microlitres of each antibody (CD34-FITC, CD90-APC, STRO-1-PE, CD146-PerCP-Cy5.5) (Thermo Fisher, USA), and 50 µL of cell suspension were added together for 20 min at 25 °C to confirm PDLSCs purity. Then, the expression of a surface marker in PDLSCs was analysed by a FACSCanto machine (BD Biosciences, USA).

### Osteogenic and adipogenic induction

To identify the osteogenic differentiation potential of PDLSCs, the cells (P3) were plated into 6-well plates at a density of 2 × 10^4^ cells per well at 37 °C in a humidified atmosphere of 5% CO_2_. Once the cells reached 60%~70% confluence, the medium was changed to an osteogenic-inducing medium (Cyagen, USA). After induction for two to four weeks, the cells were fixed with paraformaldehyde for 30 min and stained with Alizarin Red-S (Beyotime, China) for 3 ~ 5 min.

To identify the adipogenesis differentiation potential of PDLSCs, the cells (P3) were plated into 6-well plates at a density of 2 × 10^4^ cells per well at 37℃ in a humidified atmosphere of 5% CO_2_. Once the cells reached 100% confluence, the medium was changed to adipogenic inducing medium (Cyagen, USA). After induction for three to four weeks, the cells were fixed with paraformaldehyde for 30 min and stained with Oil Red O for 30 min.

### CCK-8 assays

A Cell Counting Kit-8 (CCK-8; Fude, China) was used to measure the effects of GQDs on the proliferation of PDLSCs. PDLSCs at the 2nd passage (P2) were seeded into a 96-well plate (3 × 10^4^ cells/mL). After being cultured with complete medium for 24 h, the medium was changed to complete medium containing GQDs (XFNAN, China) at various concentrations (0; 5; 10; 15; 20; 25; and 30 µg/mL) using three duplicate wells for each concentration group. Through the addition of 10 µL CCK-8 solution for 3 h at 37 °C in a dark room, assays were performed at 0, 1, 3, 5 and 7 days, and absorbance values were detected at 450 nm.

### Osteogenic differentiation of PDLSCs by GQDs

To explore the effects of GQDs and osteogenic medium and cotreatment on the differentiation of PDLSCs, PDLSCs were cultured as described above. According to our previous study (Be ready for publication), when the concentration of GQDs is 5 and 10 µg/mL, the PDLSCs migration speed is the strongest. Thus, in this work, the GQDs concentration was set as 5 µg/mL and the TNF-α concentration was set as 10 µg/mL [32]. The study groups were set as follows: (i) N + osteo group (osteogenic inducing medium without TNF-α treatment as the control group); (ii) osteo group (osteogenic inducing medium with TNF-α treatment); (iii) 5GQDs group (complete medium with 5 µg/mL GQDs and TNF-α cotreatment); and (iv) 5GQDs + osteo group (osteogenic inducing medium with 5 µg/mL GQDs and TNF-α cotreatment).

### Total RNA isolation and gene expression analysis

The mRNA expression levels of alkaline phosphatase (ALP), runt-related transcription factor 2 (RUNX2), osteocalcin (OCN), type I collagen (COL-I), low-density lipoprotein receptor-related protein-6 (LRP6), β-catenin, and lymphoid enhancer factor-1 (LEF1) were measured by qPCR analysis. Total RNA (1000 ng) was extracted using TRIzol reagent (Chuandong, China) and reverse transcribed into cDNA with HiScript III qRT SuperMix (Vazyme, China). Then, qPCR analysis was conducted using a QuantStudio5 (Thermo Fisher, USA), and the mRNA expression levels were analysed with the comparative 2^−ΔΔCt^ method. Glyceraldehyde-3-phosphate dehydrogenase (GAPDH) was used as the housekeeping gene, and the primer sequences used in the present study are listed in Table 1.

**Table 1:** Primer sequences of each gene

Name	Forward	Reverse
GAPDH	TCTCCTCTGACTTCAACAGCGACA	CCCTGTTGCTGTAGCCAAATTCGT
RUNX2	CCACTGAACCAAAAAGAAATCCC	GAAAACAACACATAGCCAAACGC
ALP	AGCTTCAAACCGAGATACAAGCA	CTGTTCAGCTCGTACTGCAT
OCN	CCCAGTCCCCTACCCGGAT	AGCAGAGCGACACCCTAGACC
LRP6	ACGCATTTCTTTGGATACACC	ACAGGATCGTAATCTATGGCAA
β-catenin	GCCTGGTTTGATACTGACCT	ACAAATAGCCTAAACCACTCCC
LEF1	AAGAGGAAGGCGATTTAGCTG	TCCTGAGAGGTTTGTGCTTGT

### Alizarin red-S staining

Alizarin red-S staining was applied to assess the formation of mineralized nodules. Stem cells (P3) were cultured in 24-well plates for 14 days and rinsed with PBS to remove the residual medium. Then, the cells were fixed with 4% paraformaldehyde (Solarbio, China) for 20 min and stained with Alizarin red-S for 30 min. Finally, the stained plates were observed with an inverted microscope (Nikon TS2, Japan) and photographed to evaluate the mineralized nodules.

### Statistical analysis

SPSS 26.0 software and GraphPad Prism 9 were applied for statistical analysis. All data are reported as the mean ± standard deviation (SD). One-way ANOVA (when the square difference was homogeneous) or nonparametric tests (when the square difference was uneven) were used to analyse statistical significance between the groups, with a *p* value < 0.05 indicating a significant difference. All experiments were repeated in triplicate.

## Results

### Cultivation and characterization of PDLSCs

**Fig. 1:**
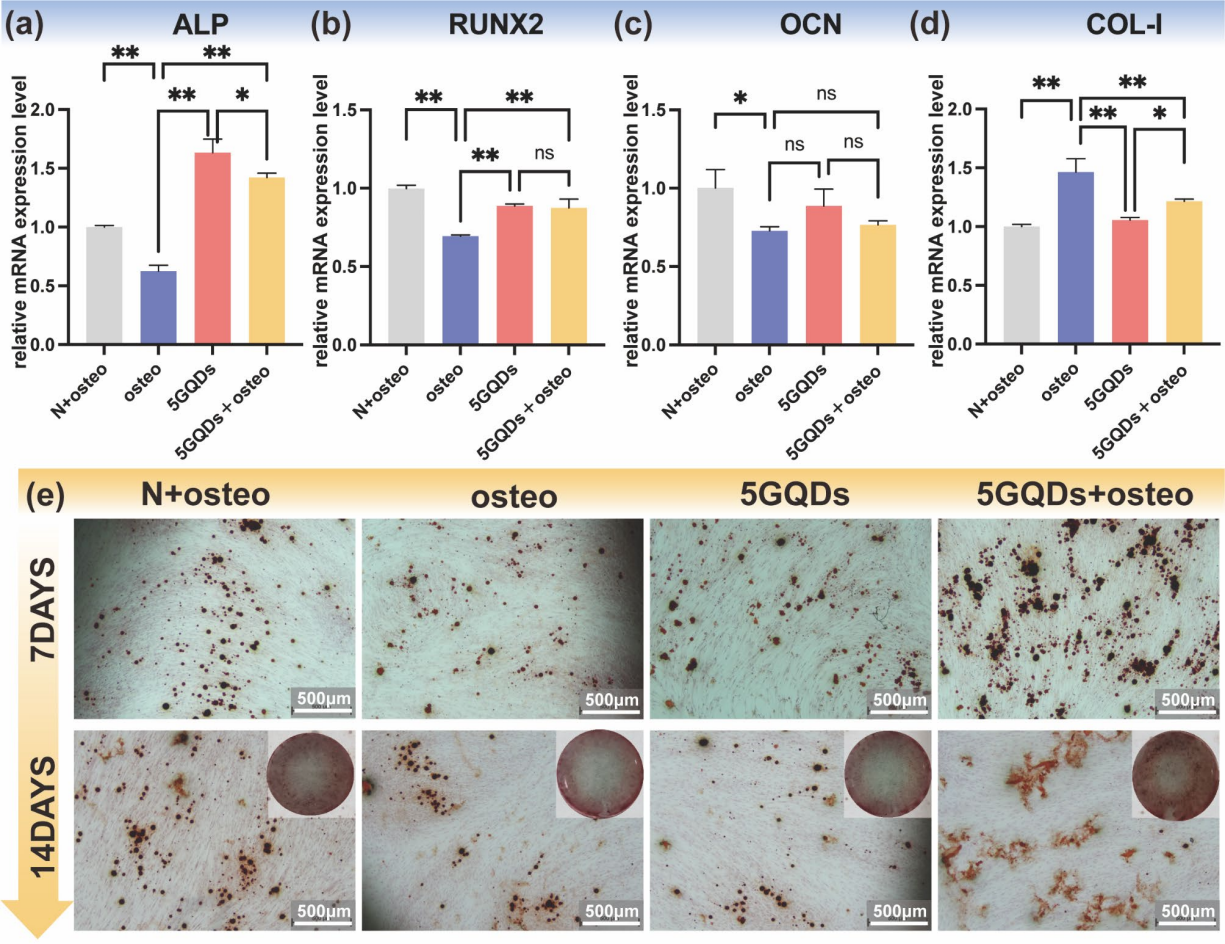
Morphological and molecular characteristics of isolated PDLSCs. (**a-b**) microphotography of alive primary periodontal ligament cells (**a**) magnification 40×; (**b**) magnification 100×. (**c**) colony culture of PDLSCs and single-cell colony culture (inset panel). (**d**) microphotography of PDLSCs colony stained with Crystal Violet. (**e**) microphotography of representative data for Alizarin red-S staining of PDLSCs differentiates into osteoblasts. (**f**) microphotography of representative data for Oil Red O staining of PDLSCs differentiates into adipocyte. (**g-j**) flow cytometry was used to analyse the surface markers of PDLSCs; Positive rate: (**g**) CD90-100%, (**h**) CD146- 90.4%, (**i**) STRO-1-82.7%, (**j**) CD34-0.5%.

PDLSCs were obtained and cultured with the tissue block method, and primary cells grew from the edge of the tissue and proliferated around the tissue mass after 7 to 14 days. The morphology of PDLSCs was similar to that of fibroblasts with elongated shapes (Fig. 1a, b). Then, PDLSCs were isolated and purified in a 96-well plate with the limited dilution method, adjusting the concentration so that there was only one cell per well (Fig. 1c inset panel). Following monoclonal culture, PDLSCs showed a long shuttle shape and swirling growth with directionality and regularity (Fig. 1c-d). To characterize the MSC properties of these cells, flow cytometry analysis was conducted to identify the expression levels of surface markers. The results showed that MSC phenotypic molecular markers were expressed on the PDLSCs, including CD90 (100%), CD146 (90.4%), and STRO-1 (82.7%), while the haematopoietic marker CD34 (0.5%) was not observed (Fig. 1g-j). Furthermore, calcified nodules were shown in the Alizarin red-S staining test, demonstrating that PDLSCs had the potential to differentiate into osteoblasts (Fig. 1e). Lipid droplet formation was observed in the Oil red O staining test, revealing that PDLSCs had differentiated into adipocytes (Fig. 1f).

### Effect of GQDs on the proliferation of PDLSCs

**Fig. 2:**
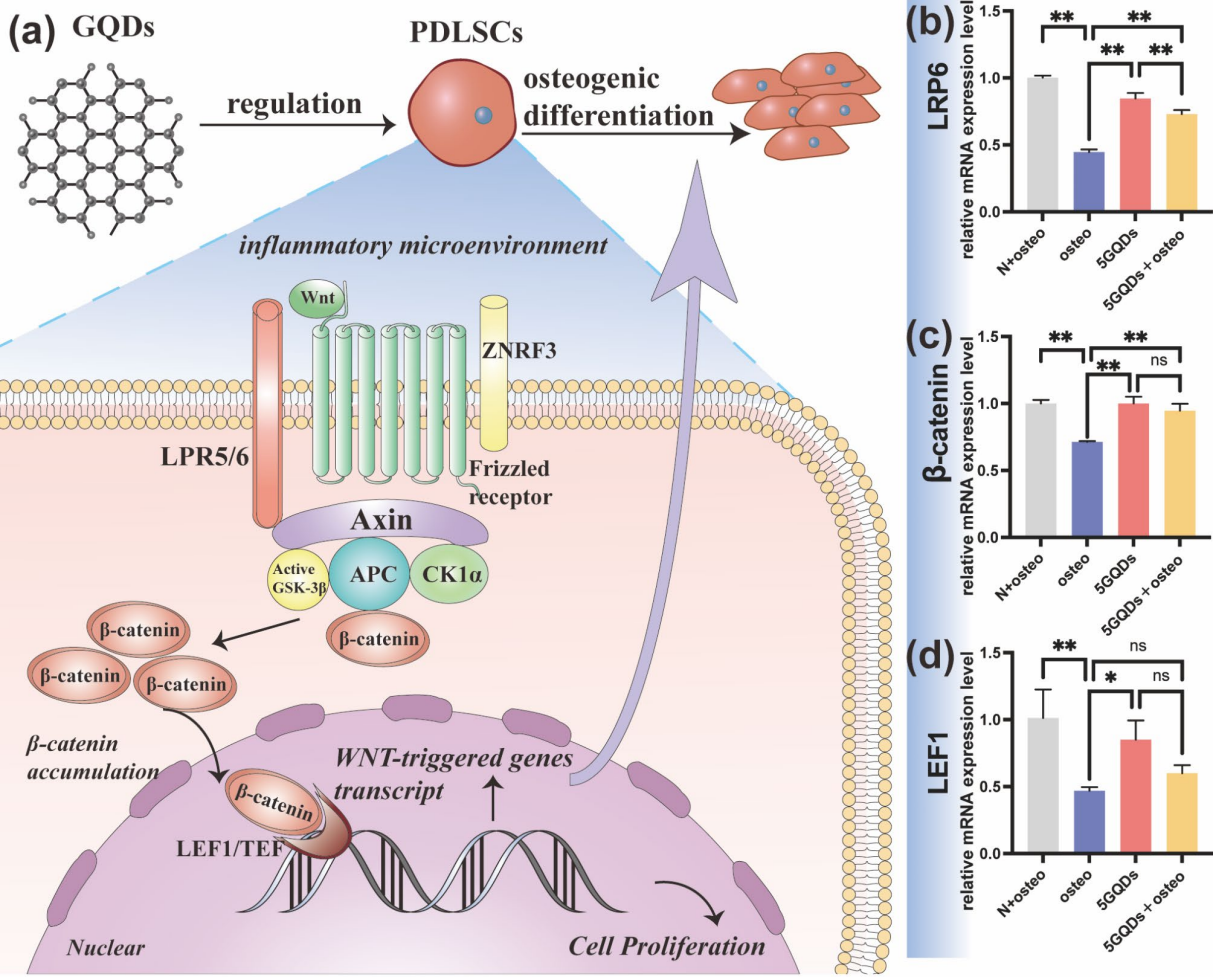
Effects of different concentrations (0–30 µg/mL) of GQDs on the proliferation of PDLSCs under the media mimicking proinflammatory environment and the standard media as the control group. (SM-standard media, IM-inflammatory microenvironment)

To explore the effect of GQDs on the proliferation of PDLSCs under TNF-α-induced inflammatory microenvironments (IM) and standard medium (SM) as the control group, CCK-8 assays were performed. As shown in Fig. 2, from Day 0 to Day 7, PDLSCs proliferation significantly increased at concentrations of 0 ~ 30 µg/mL GQDs (*p* < 0.05). Except for Day 3, there are no significant difference in proliferation rate was observed between groups within the same day (*p* > 0.05). Specifically, there is significant difference on Day 3 between the concentration group of 0 µg/mL and 10 µg/mL GQDs under the IM mimicking medium, as well as the group of 0 µg/mL and 15 µg/mL and the group of 0 µg/mL and 25 µg/mL. Moreover, TNF-α (10 µg/mL) and GQDs cotreatment had no significant effect on the proliferative ability of PDLSCs compared to the control group (SM).

### Osteogenic differentiation of PDLSCs by GQDs under an inflammatory environment

**Fig. 3:**
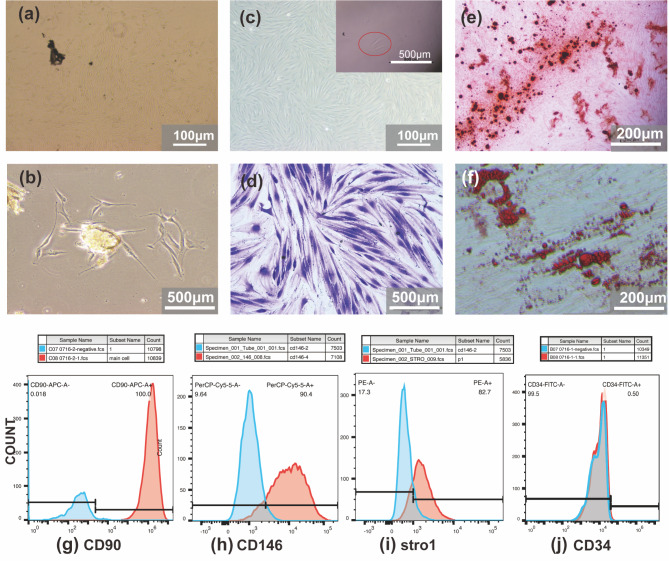
Effect of GQDs on the osteogenic differentiation of PDLSCs. (**a-d**) the expression of ALP, RUNX2, OCN, and COL-1 were detected by qRT-PCR at Day 7. Data are expressed as mean ± SD from three independent experiments performed in triplicate.*p<0.05, **p<0.01, ns: No statistical difference. (**e**) Alizarin S Red staining was performed to detect the mineralized nodules on Day 7 and Day 14

After treatment with 5 µg/mL GQDs and 10 µg/mL TNF-α for 7 days, the results showed that TNF-α treatment decreased the expression of osteogenic genes (Fig. 3a-c). The expression levels of osteogenic-related genes (including ALP and RUNX) significantly increased in the 5GQDs and 5GQDs + osteo groups at Day 7 compared to the osteo group (Fig. 3a-b). For OCN, the increasing trend was not statistically significant (Fig. 3c). Nevertheless, cellular exposure to either the 5GQDs group or the 5GQDs + osteo group for 7 days resulted in a decrease in the mRNA levels of COL-I compared with the osteo group (Fig. 3d).

### GQDs enhance mineralized nodule formation in PDLSCs

As a late marker of osteogenic differentiation, mineralized nodules were detected by Alizarin red-S staining to estimate the part played by GQDs on PDLSCs. Compared with the control group (N + osteo group), mineralized nodule formation decreased in the osteo group. Following GQDs treatment, the formation of mineralized nodules strongly increased in the presence of TNF-α, particularly on Day 7 and Day 14 (Fig. 3e).

### Osteogenic differentiation of PDLSCs by GQDs via the Wnt/β-catenin signalling pathway

**Fig. 4:**
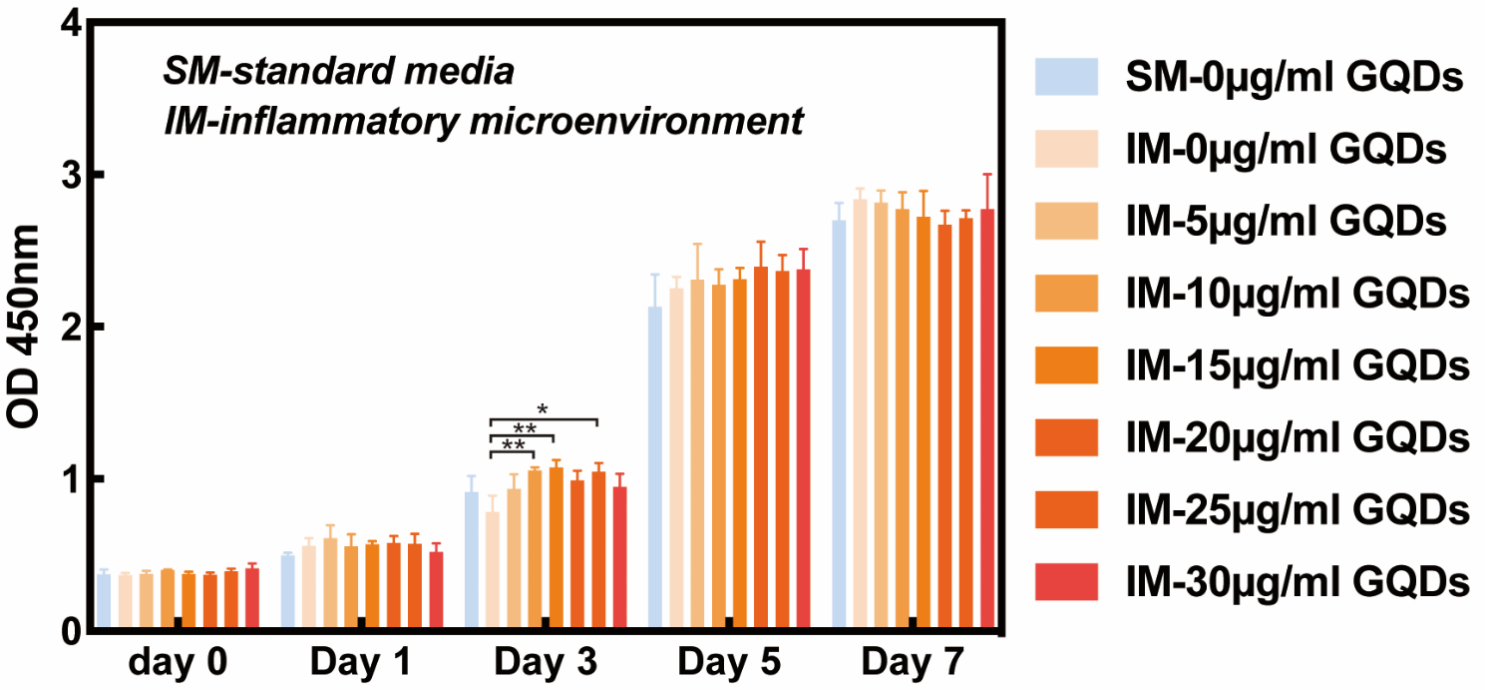
(**a**)Schematic demonstrated that GQDs promote osteogenic differentiation of PDLSCs through the Wnt/β-catenin signalling pathway. (**b**) LRP6 expression (**c**) β-catenin and (**d**) LEF1 were assessed by qRT-PCR on Day 7. *p<0.05, **p<0.01, ns: No statistical difference

The mechanism by which GQDs facilitate the osteogenic differentiation of PDLSCs is unknown. However, we found in the literature that the Wnt/β-catenin signalling pathway is involved in the osteogenic differentiation of PDLSCs. Therefore, to explore the underlying mechanism by which GQDs promote PDLSCs osteogenic differentiation, Wnt signalling pathway-related genes, including LRP6, β-catenin, and LEF1, were analysed. Compared to the control groups (N + osteo group), TNF-β significantly decreased the expression of Wnt signalling pathway-related genes in the osteo group. The expression levels of LRP6 and β-catenin were significantly increased in the 5GQDs and 5GQDs + osteo groups at Day 7 compared to the osteo group (Fig. 4b-c). In addition, LEF1 levels were elevated in the 5GQDs and 5GQDs + osteo groups at Day 7, but the elevation in the 5GQDs + osteo group was not statistically significant (Fig. 4d).

## Discussion

The ideal treatment for periodontitis is to successfully reverse the inflammatory microenvironment and achieve periodontal tissue reconstruction. PDLSCs are dependable seed cells for repairing periodontal defects because of their multidirectional differentiation potential, easy accessibility, and strong periodontal correlation. However, the inflammatory microenvironment could suppress the proliferation and differentiation capability of PDLSCs. Therefore, a variety of novel biomaterials have been explored for application in periodontal tissue engineering to mimic the growth microenvironment and enhance the multidirectional differentiation potential. In this study, GQDs, novel graphene nanomaterials, were first employed to stimulate PDLSCs to modulate cellular behaviours, including proliferation and osteogenic differentiation, and the results showed that GQDs could enhance the osteogenic differentiation capability, we speculated that regulated by activating the Wnt/β-catenin signalling pathway.

As a zero-dimensional structure of graphene nanomaterials, GQDs are characterized by their chemical inertness and compatible functionalization with biomolecules and could act as promising candidates for periodontal tissue engineering [33]. However, biosafety issues and the optimum working concentration to facilitate biological behaviours have not yet been clarified. Previous toxicity research reported that the inhalation of graphene and graphene oxide (GO) induced minimal pulmonary toxicity, but bolus airway exposure to graphene and GO caused acute and subacute pulmonary inflammation [34]. Nevertheless, due to their ultrasmall size, high oxygen content, and easy metabolism, the cytotoxicity of GQDs is lower than those of GO and rGO. A photoluminescence study showed that GQDs were randomly distributed in the cytoplasm and did not diffuse into the nucleus of mammalian cells such as osteoblastic cells (MC3T3-E1), suggesting that GQDs have low toxicity to mammalian cells [35, 36]. The in vivo experiment of GQDs also revealed that there was no material accumulation in the main organs of mice and that the clearance of GQDs through the kidney was fast [37]. These results were consistent with our findings, which confirmed that the treatment of GQDs had no significant effect on the proliferative ability of PDLSCs, suggesting their good biocompatibility and low toxicity.

Compared with the biological interface behavior and non-covalent binding of graphene material make it obtain the ability to absorb osteoinductive factors to accelerate MSCs osteogenic differentiation [38, 39], GOQDs lies in its ultrasmall size and good biocompatibility, may interact with extracellular matrix (ECM) and ingested by cells to influence MSCs differentiation [24, 28]. Now, it is generally accepted that the upregulation of ALP expression levels, as well as mineralized nodules, are considered early indicators for identifying the osteogenic differentiation potential of cells [40, 41]. ALP, an enzyme secreted by osteoblasts, can promote the deposition of calcium ions in collagen to complete matrix mineralization [41]. In this study, we found that GQDs could promote the expression of ALP, and the expression level was higher than that of the group with the added osteogenic-inducing medium. These results were reconfirmed by Alizarin red-S staining, demonstrating that GQDs could spontaneously promote osteogenic differentiation of PDLSCs without the addition of chemical inducers.

RUNX2, COL-I, and OCN are also strongly related to the osteogenic differentiation of PDLSCs and were selected to assess osteogenic differentiation by qRT‒PCR, with GAPDH as the standard reference. RUNX2, an essential transcription factor for osteogenic differentiation, not only enhances the proliferation of suture mesenchymal cells and induces their commitment into osteoblast lineage cells but also induces the expression of major bone matrix protein genes, including COL-I and OCN [42, 43]. COL-I, secreted by osteoblasts, is the primary component of the ECM and serves as a scaffold upon which minerals are deposited [44]. Furthermore, OCN, the most abundant noncollagenous protein in bone tissue, is involved in the control of the mineralization process and expressed in mature bone-forming cells at a late stage of osteogenic differentiation [45, 46]. In our study, the inflammatory microenvironment suppressed osteogenic-related gene expression, but the mRNA expression levels of RUNX2 in PDLSCs were upregulated by GQDs treatment. Therefore, this finding could be the basis for the experimental clinical application of GQDs for periodontal tissue regeneration.

Although GQDs treatment has proven to be effective in bone regeneration, the molecular mechanism by which GQDs could promote the osteogenic potential of PDLSCs is unknown. There is definite evidence that the Wnt/β-catenin signalling pathway is involved in regulating a variety of biological behaviours and plays a crucial role in osteogenic differentiation through the up-regulation of RUNX2 [31, 47, 48]. When the signalling pathway is activated, the Wnt ligand binds to frizzled receptors and LRP5/6 to form a complex and subsequently binds to the Axin/GSK-3β/APC complex, which reduces its degradation activity and stabilizes β-catenin in the cytoplasm. The accumulated β-catenin will thereby enter the nucleus, bind to LEF/TCF, and stimulate transcription of the downstream target gene, which will promote the expression of osteogenic-related factors, such as ALP (Fig. 4a). It is generally known that the Wnt/β-catenin pathway plays essential roles in osteogenesis and homeostasis through the upregulation of Runx2 [48]. Interestingly, GOQDs could upregulate β-catenin expression in SHED and BMSCs to promote osteogenesis [24, 49]. In addition, exposure to 50 µg/mL maGO-CaP in a coculture of hMSCs and monocytes upregulated LEF1 expression and thereby stimulated bone formation in vivo [50]. During periodontitis, the expression levels of LRP6, β-catenin, and LEF1 were suppressed in the inflammatory microenvironment. The expression levels of Wnt signalling pathway-related genes were higher in the 5GQDs and 5GQDs + osteo groups than in the osteo group. This result suggested that GQDs might activate the Wnt/β-catenin singalling pathway to osteogenic differentiation of PDLSCs, but the mechanism needs to be further confirmed by subsequent western blot analysis and mRNA chips.

Taken together, our research proved that TNF-α inhibits the capacity of osteogenic differentiation of PDLSCs. At appropriate concentrations, GQDs have no biotoxicity and might promote the osteogenic differentiation of PDLSCs by initiating the Wnt/β-catenin signalling pathway. These findings might provide a scientific basis for the application of carbon-based nanomaterials in periodontal tissue engineering. However, the exact mechanism of the effect of GQDs on PDLSCs is not fully understood, and more studies, including western blot analysis and in vivo experiments, are needed to clarify the biological action of GQDs on PDLSCs. Here, we deem that GQDs could serve as an osteoinductive scaffold, recruiting native cells to the site of injury and promoting differentiation into bone cells.

## Data Availability

All data generated or analyzed during this study are included in this published article.
