# Correction: Graphene quantum dots enhance the osteogenic differentiation of PDLSCs in the inflammatory microenvironment

**DOI:** 10.1186/s12903-026-07920-8

**Published:** 2026-03-31

**Authors:** Wanshan Gao, Yan Liang, Dongyan Wu, Sicheng Deng, Rongmin Qiu

**Affiliations:** 1 College of Stomatology, Hospital of Stomatology Guangxi Medical University , Guangxi Key Laboratory of Oral and Maxillofacial Rehabilitation and Reconstruction, Guangxi Clinical Research Center for Craniofacial Deformity, Guangxi Key Laboratory of Oral and Maxillofacial Surgery Disease Treatment, Guangxi Health Commission Key Laboratory of Prevention and Treatment for Oral Infectious Diseases, Nanning, Guangxi 530021 China; 2 Key Laboratory of Research and Application of Stomatological Equipment College of Stomatology Hospital of Stomatology Guangxi Medical University, Education Department of Guangxi Zhuang Autonomous Region, Nanning, Guangxi 530021 China

**Correction to: BMC Oral Health 23, 331 (2023)**

**https://doi.org/10.1186/s12903-023-03026-7**

In this article [1], the Figs. 1, 2, 3 and 4 were wrongly numbered as Figs. 3, 4, 1 and 2 respectively. The correct figures with their caption are provided in this correction.

**Fig. 1:**
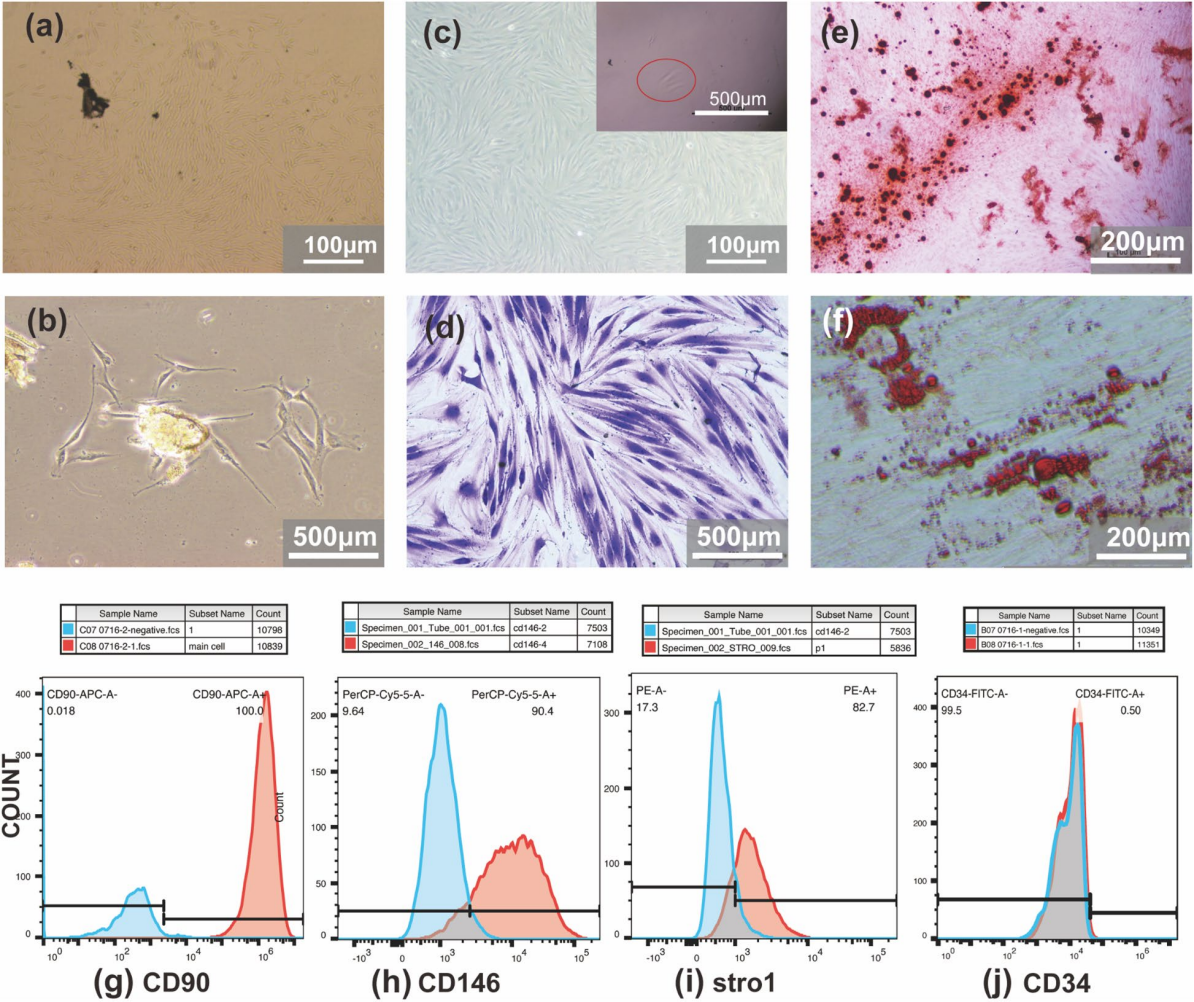
Morphological and molecular characteristics of isolated PDLSCs. **a**, **b** microphotography of alive primary periodontal ligament cells (a) magnification 40×; (b) magnification 100×. **c** colony culture of PDLSCs and single-cell colony culture (inset panel). **d** microphotography of PDLSCs colony stained with Crystal Violet. **e** microphotography of representative data for Alizarin red-S staining of PDLSCs differentiates into osteoblasts. **f** microphotography of representative data for Oil Red O staining of PDLSCs differentiates into adipocyte. **g**-**j** flow cytometry was used to analyse the surface markers of PDLSCs; Positive rate: (g) CD90-100%, (h) CD146- 90.4%, (i) STRO-1-82.7%, (j) CD34-0.5%

**Fig. 2:**
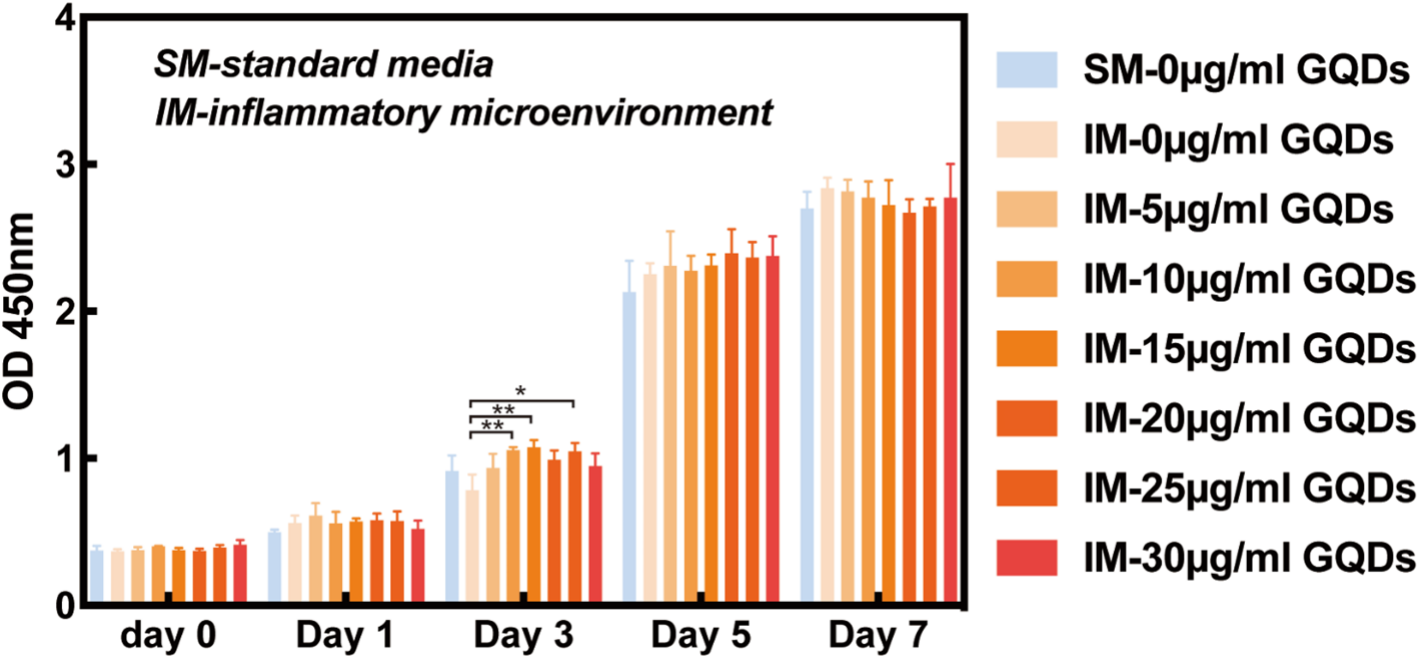
Effects of different concentrations (0–30 µg/mL) of GQDs on the proliferation of PDLSCs under the media mimicking proinflammatory environment and the standard media as the control group. (SM-standard media, IM-inflammatory microenvironment

**Fig. 3:**
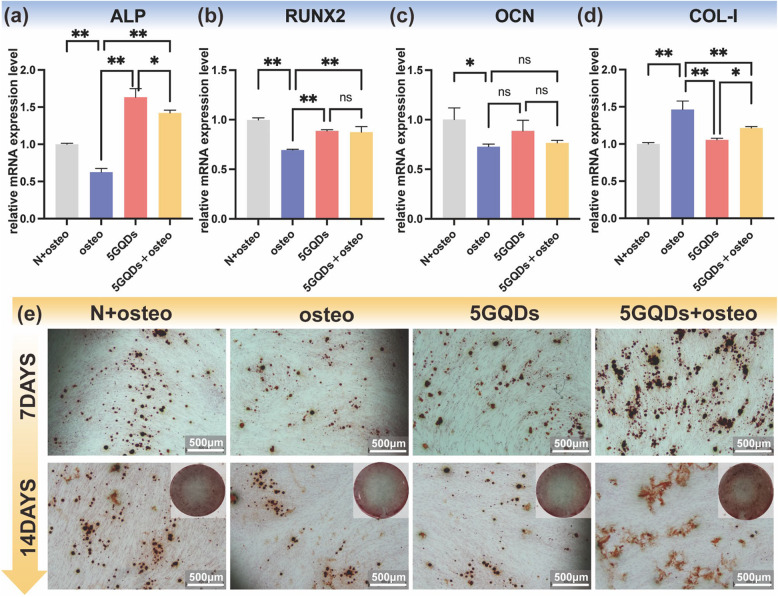
Effect of GQDs on the osteogenic differentiation of PDLSCs. **a**-**d** the expression of ALP, RUNX2, OCN, and COL-1 were detected by qRT-PCR at Day 7. Data are expressed as mean±SD from three independent experiments performed in triplicate.*p<0.01 , ns: No statistical difference. **e** Alizarin S Red staining was performed to detect the mineralized nodules on Day 7 and Day 14

**Fig. 4:**
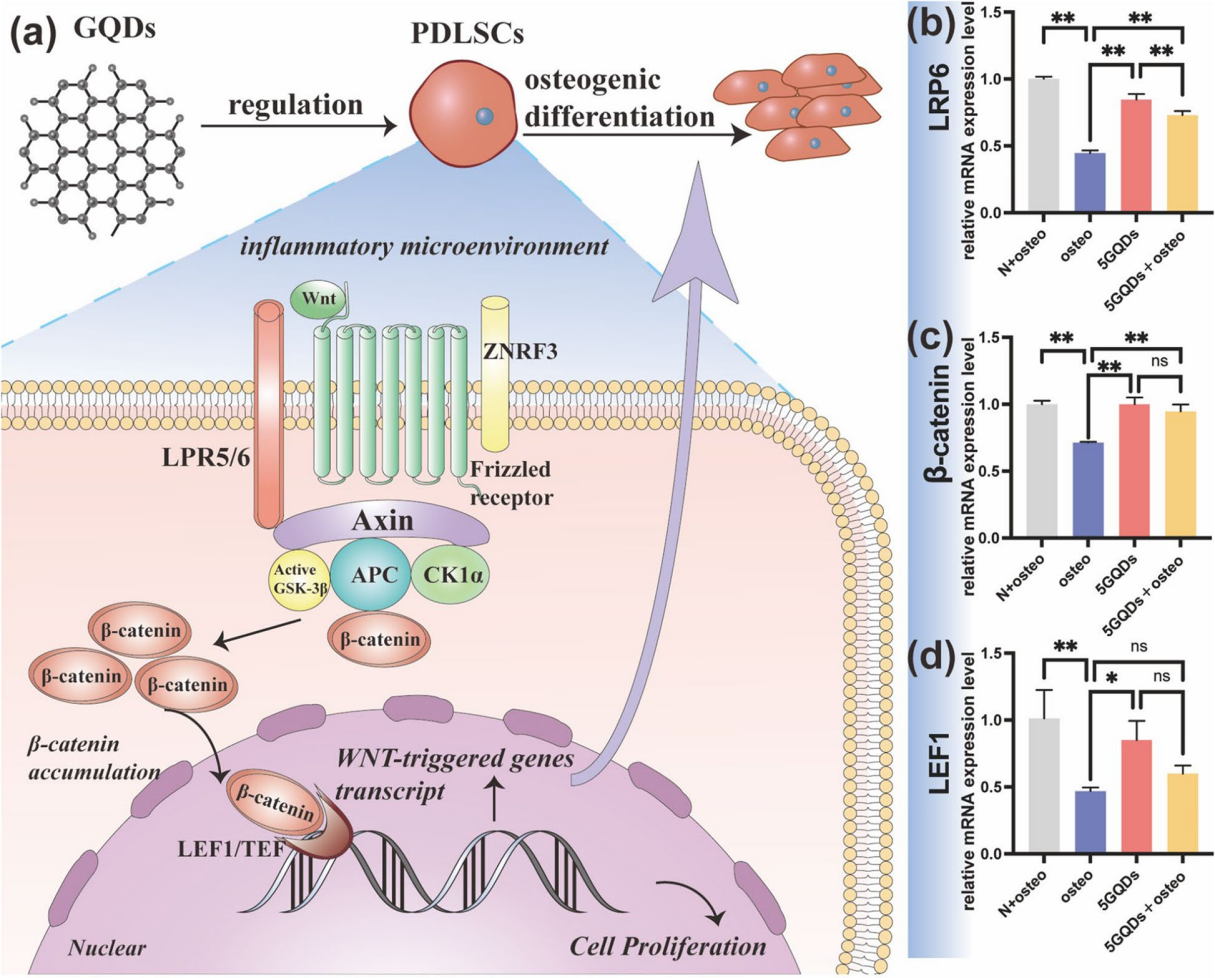
**a** Schematic demonstrated that GQDs promote osteogenic differentiation of PDLSCs through the Wnt/β-catenin signalling pathway. **b** LRP6 expression (**c**) β-catenin and (**d**) LEF1 were assessed by qRT-PCR on Day 7. *p<0.05, **p<0.01,ns: No statistical difference
